# Properties of Concrete Prepared with Recycled Aggregates Treated by Bio-Deposition Adding Oxygen Release Compound

**DOI:** 10.3390/ma12132147

**Published:** 2019-07-03

**Authors:** Yaguang Zhu, Quanquan Li, Peizhen Xu, Xiangrui Wang, Shicong Kou

**Affiliations:** 1 School of Civil Engineering, Qingdao university of technology, Qingdao 266033, China; 2 Guangdong Provincial Key Laboratory of Durability for Marine Civil Engineering, Shenzhen University, Shenzhen 518060, China

**Keywords:** recycled aggregate, bio-deposition treatment, oxygen release compound, compressive strength

## Abstract

Recycled aggregates have high water absorption and crushing index. In order to improve the properties of recycled aggregates in concrete production, various treatments were used to modify the aggregates. In recent years, bio-deposition as a new treatment method of recycled aggregates was environmentally friendly. An improved method of bio-deposition was implemented to modify the properties of recycled mortar aggregates (RMA). O-bio-deposition is based on aerobic bacteria induced CaCO_3_ precipitation by respiration by varying the distance between the RMA and the bottom of the container and by adding an oxygen release compound to the culture solution that contains bacteria to promote the induction of CaCO_3_. First, the physical properties, including water absorption, crushing value, and apparent density, of the coarse RMA under different treatment methods were determined, and an o-bio-deposition treatment method was obtained. The fine RMA was treated and compared with the untreated RMA. Concretes were then prepared from the treated coarse RMA, and compressive strength and slump were determined. In addition, the effect of the o-bio-deposition treatment on the RMA surface and the micro-cracks of concretes were observed by scanning electron microscopy (SEM). It was found that the water absorption and crushing index of the coarse RMA treated by o-bio-deposition were reduced by 40.38 and 19.76% compared with untreated RMA, respectively. Regarding the concrete, the slump and the compressive strength (28 d) of concrete were increased by 115% and 25.3%, respectively compared with the untreated concrete.

## 1. Introduction

In China, with the rapid development of the construction industry, the amount of construction and demolition (C&D) waste produced increases. Approximately 20 billion tons of waste concrete were produced annually in 2017 [1]. From the perspective of sustainable development, recycling waste concrete to produce recycled concrete aggregate (RCA) can effectively reduce harm to the environment [2,3]. However, the attached mortar on the surface of recycled aggregates leads to higher water absorption and crushing value of RCA [4,5,6,7]. Verian et al. [8] reported that the water absorption of natural aggregates (NA) ranged from 0.34%–3.00% and RCA ranged from 0.50%–14.75%. Saravanshi et al. [6] reported that the crushing value for RCA is 33% higher than that of NA. Therefore, the compressive strength and durability of recycled aggregate concrete (RAC) prepared with RCA were negatively affected. [7,9,10,11,12,13,14]; the compressive strength of RAC for 100% aggregates replaced by RCA can decrease by about 30% compared to concrete made with NA [5,8]. Currently, researchers are committed to modifying recycled aggregates to improve the mechanical properties of recycled aggregates and propose methods for this.

Among previous studies, researchers reported numerous results regarding the improvement of RCA. The main improving measures have included removing and strengthening the adherent mortar [15]. Mechanical grinding is a simple and efficient method that is widely used to remove the adherent mortar, where the water absorption of treated RCA decreased by about 50% and the improvement in compressive strength of concrete at 28 d was 39% [16]. Moreover, Sui et al. [17] heated RCA first, to weaken the adhered mortar as a result of the generated thermal stresses. Then RCA was ground by a machine. The combination of heating and grinding treatment improved the apparent density of RCA by 4.67% at 600 °C. However, owing to the collisions between the machinery and the aggregate, numerous new micro-cracks are produced, which damage the RCA. Tam et al. [18] used three kinds of acid solutions to immerse RCA in, and they reported that the water absorption reduced by 12% after hydrochloric acid (HCl) solution treatment. Saravanakumar et al. [6] treated HCl-treated RCA with silica fume by immersing it in silica fume solution, the compressive strength at 28 d improved by 22% for treated aggregate concrete. However, this HCl treatment causes secondary pollution to the environment [16]. Therefore, Wang et al. [19] immersed RCA in a safe and environmentally friendly solution of acetic acid and then ground RCA by a machine. After treatment, the water absorption of RCA reduced by 19%.

As an alternative, strengthening the adhered mortar is also valuable for the reinforcement of RCA. Polymer impregnation is very effective for reducing water absorption and is often used in RCA modification [20,21,22]. Polymer particles can be filled in the pores of RCA and can form a waterproof coating on the surface of the RCA. Kou and Poon [21] treated RCA with a PVA (polyvinyl alcohol) solution, and the water absorption of RCA after the treatment significantly reduced by up to 79% (for a particle size of 20 mm). Spaeth and Tegguer [22] reported that the water absorption of RCA decreased by at least 50% upon the use of siloxane and silane polymer treatments. Wang et al. [23] immersed RCA in a diammonium hydrogen phosphate (DAP) solution. As a result, the water absorption of the aggregate evidently decreased by 18.2%, and the compressive strength of concrete for 14 d increased by 18.9%. Carbonation treatment of RCA is an environmentally friendly approach [12]. CO_2_ can react with C-S-H gel and calcium hydroxide to form calcium carbonate precipitation, which fills the voids and pores of the adhered mortar [24]. Kou et al. [25] showed that the water absorption of the recycled mortar aggregate (RMA) significantly improved (38%) and the compressive strength of the concrete showed little improvement with this carbonation treatment. The RMA obtained from the crushing mortar blocks was used to study the improvement of the attached mortar on RCA after treatment in this study.

Recently, ‘bio-deposition’ has been widely reported as a novel environmentally friendly method for modifying RCA by strengthening the adhered mortar. Currently, researchers are mainly focused on the bio-deposition in concrete cracks [26,27,28,29]. For RCA, this method is based on the ability of bacteria to induce calcium carbonate deposition in a calcium-rich environment to fill or bind micro-cracks of RCA [30,31,32,33,34]. In the past, ureolytic bacteria were most commonly applied by researchers; this involved decomposition of CO(NH_2_)_2_ into CO_3_^2−^ and NH_4_^+^ and then precipitation of CaCO_3_ in a calcium-rich environment. Wang et al. [30] designed several treatment methods based on spraying and immersion methods of bio-deposition. The results showed that the weight increased by 2% and the decrease in water absorption of the RCA was 10% after two immersion treatments. Qiu et al. [31] reported that the water absorption reduction of RCA using bio-deposition was 15% and the weight of RCA increased 0.3% under certain conditions of pH, temperature, bacterial concentration, and Ca^2+^ concentration. However, ammonia produced by ureolytic bacteria not only caused corrosion of the steel bars but also polluted the environment [26]. Therefore, Wu et al. [34] used the bio-deposition method, which was based on Bacillus pseudofirmus (DSM8715) to induce CaCO_3_ precipitation by respiration. The results showed that the water absorption and crushing index of the RCA treated by bio-deposition both decreased (10% and 15%, respectively).

In this study, regarding the selection of strains and the treatment method of bio-deposition, Zhang et al. [35] determined and screened a strain of H4 with the highest calcium precipitation activity (CPA) of 94.8% to self-heal concrete cracks. Furthermore, Zhang et al. [36] developed an oxygen-releasing compound (ORC) that contained calcium peroxide (CaO_2_) and lactic acid that supplied oxygen (O_2_) for the calcium precipitation of the H4 strain upon contact with water. In the presence of oxygen, strain H4 induced 50% more calcium precipitation than that obtained without oxygen. Therefore, in this paper, Bacillusal alcalophilus (H4) [35] was used to modify RMA. To supply a considerable amount of O_2_ to the H4 strain to metabolize and produce CO_2_ combined with Ca^2+^ to induce CaCO_3,_ based on the above research, calcium peroxide (CaO_2_) was innovatively added to the bacterial solution as an ORC [36]. For the first time, the effect of the distance between the aggregates and the bacteria in the container on the improvement was investigated by changing the position of the RMA in the container.

First, the effects of the concentration of CaO_2_ in the culture solution on the respiration of aerobic strain H4 and the position of the RMA immersed in the container were considered; the optimum concentration of CaO_2_ and the position of RMA immersion in the container were determined by testing the water absorption, apparent density, and crushing index of the coarse RMA under different treatment methods, and the optimal treatment method of o-bio-deposition was obtained. Then, fine RMA was treated with bio-deposition and o-bio-deposition, and the characteristics of the RMAs with different particle sizes were obtained and compared with those of the untreated RMA. Finally, concrete was prepared with treated and untreated coarse RMAs, respectively, and the slump of fresh concrete and the compressive strength of hardened concrete were obtained. Furthermore, SEM was used to observe the surface of the RMA and the micro-cracks of the concrete.

## 2. Materials and Methods

### 2.1. Materials

#### 2.1.1. Recycled Mortar Aggregates

The RMA for the study was obtained from old mortar blocks. Based on the mixture ratio in GB/T 17671-1999 [37], an old cement mortar mixture was prepared with a sand to cement ratio of 2.5 and a water to cement ratio of 0.5. The mortar mixture was cast in steel molds. After 24 h, the specimens were demolded and maintained in a water curing tank at 20 ± 2 °C for 90 days. After curing, all specimens were removed from the tank and crushed using a jaw crusher, and the coarse aggregates (5–20 mm) and the fine aggregates (<5 mm) were obtained.

#### 2.1.2. Bacterial Strain and Cultivation

Bacillus alcalophilus H4 (Shenzhen University, Shenzhen, China) was used as the experimental bacteria in this study [35]. The original bacterial solution (1 × 10^8^ cells/mL) was mixed with a culture medium containing sodium lactate (10 mL/L), calcium chloride (3.33 g/L), sodium nitrate (2 g/L), and CAPS( 3-(cyclohexylamino)-1-propanesulfonic acid) (22.13 g/L) at a volume ratio of 1:9 [34].

#### 2.1.3. Cement

Ordinary Portland cement with a strength grade of P.O 42.5 was used, and the density of the cement was 3.16 g/cm^3^, whereas the specific surface area was 3519.5 cm^2^/g.

### 2.2. Bio-Deposition Treatment Method for RMA

**Figure 1:**
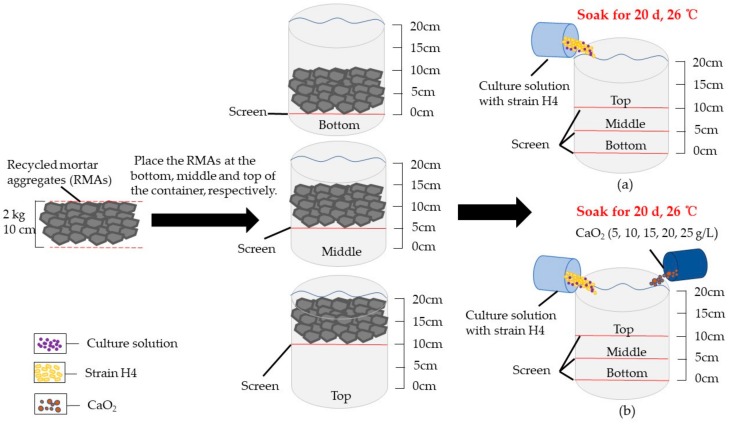
Treatment of recycled mortar aggregates (RMAs): (**a**) RMAs were immersed in the container containing bacteria solution and placed at different positions, respectively. (**b**) RMAs were immersed in the container containing bacteria solution and placed at different positions, different concentration of CaO_2_ added, respectively.

The traditional way to improve RCA is to immerse RCA in the bottom of glassware filled with a culture solution-containing bacteria (called bio-deposition) (Figure 1a). In this study, CaO_2_ was added as the ORC to provide O_2_ and a small amount of calcium, and the lactic acid powder was added to regulate the pH to 10.0. Ca(OH)_2_ is produced when CaO_2_ releases oxygen, and Ca(OH)_2_ is a micro-soluble substance; thus, the RMA was separated from CaO_2_ by a screen. The concentration of CaO_2_ added in the bacterial culture medium was set at a 5 g/L gradient, i.e., 5, 10, 15, 20, and 25 g/L.

The bio-deposition treatment of RMA occurred in φ150 × 200-mm glassware. Coarse RMA (2 kg) was placed on the top of the container (10 cm from the bottom of the container), in the middle (5 cm from the bottom of the container), and at the bottom. The culture solution with bacteria-containing CaO_2_ and a lactic acid solution was then added into the container until the liquid reached 20 cm (Figure 1b). The coarse RMA was immersed in the culture solution without mixed CaO_2_ as the control experiment (Figure 1b). The RMA was immersed at 26 °C for 20 days. The fine RMA was treated using the o-bio-deposition method, and the fine RMA was obtained from the study of the coarse RMA.

### 2.3. Test Methods

#### 2.3.1. Water Absorption

The test was performed according to the Chinese specification JGJ 52-2006 [38]. Bacillus alkalophilus H4 cannot metabolize in an environment above 60 °C [34], hence, the weight was obtained by drying at 50 °C.

#### 2.3.2. Apparent Density

JGJ 52-2006 [38] was used to determine the apparent density of RMA. RMA was dried in an oven (50 ± 5 °C) to ensure bacterial activity in concrete preparation [33].

#### 2.3.3. Crushing Index

The crushing index of the RMA was determined by the Chinese specification JGJ 52-2006 [38]. The coarse RMA with particle sizes of 10–20 mm was placed into a steel mold, and the surface was levelled and the mold was placed on the loading machine. The maximum load was 200 kN at a rate of 1 kN/s, and the load was retained for 5 s. After loading, the coarse RMA was poured out and determined (*m*_0_). The weight of the residual RMA was determined by sieving with a 2.36-mm pore size sieve (*m*_1_). The crushing index (*C*c=*C*) of coarse RMA was calculated according to Equation (1).

Fine RMA was divided into four classes of different particle sizes: 5–2.5 mm, 2.5–1.25 mm, 1.25–0.63 mm, and 0.63–0.315 mm. The fine RMA was placed into the steel mold and loaded by a loading machine at a rate of 500 N/s to 25 kN and retained for 5 s. After loading, the fine RMA was sieved through 2.5, 1.25, 0.63, and 0.315-mm pore size sieves successively. The crushing index of each class was determined using Equation (1) (*C* = *a*_1_, *a*_2_, *a*_3_, and *a*_4_); then, the total crushing index of fine RMA was determined using Equation (2) [34]. The values used were the average of three measurements.

(1) $$C=(\frac{m_{0}-m_{1}}{m_{0}})\times 100\%$$

where:

*C* = crushing index (%)
*m*_0_ = weight of the RMA (g)
*m*_1_ = weight of RMA after crushing test (g)

(2) Csa=a1c1+a2c2+a3c3+a4c4a1+a2+a3+a4×10000

where:

*C*_sa_ = The total crushing index (%) was accurate to 0.1%.
*a*_1_, *a*_2_, *a*_3_, and *a*_4_ = the crushing index of fine RMA with different particle sizes of 2.50 mm, 1.25 mm, 0.63 mm, and 0.315 mm, respectively.
*c*_1_, *c*_2_, *c*_3_, and *c*_4_ = the corresponding residual weights (%)

#### 2.3.4. Characterization of the Surface of RMA by SEM-EDS

Scanning electron microscopy (SEM) was used to observe that CaCO_3_ that was induced by bacteria filled the pores and micro-cracks of RMA. Energy dispersive spectroscopy (EDS) analysis was conducted simultaneously to obtain the chemical composition of the particles found on the surface of RMA. The effect of the treatment on the micro-cracks in concrete was observed by SEM (Gemini SEM 300/VP ultra-high resolution field emission scanning electron microscope produced by Zeiss, Oberkochen, Germany).

### 2.4. Concrete Properties

The untreated and treated coarse RMAs were the only coarse aggregates used to make concrete with the mix shown in Table 1, and they were proportioned following the Chinese specification JGJ 55-2011, GB/T 25177–2010 [39,40]. The coarse RMA treated with the optimal treatment method of o-bio-deposition was used for making concrete (denoted as O-RMA/C). The coarse RMA treated by bio-deposition was used to make a control concrete (denoted as B-RMA/C) to examine the effect of the bio-deposition treatment. In addition, untreated coarse RMA was used to prepare concrete (denoted as U-RMA/C) as an extra reference. However, owing to the different water absorption of the aggregates, additional water was used to keep the saturated surface of the aggregates dry before mixing; thus, the actual total amount of water is shown in Table 1. The concrete mixture was poured in a 100 mm × 100 mm × 100 mm steel mold. After 48 h, the specimens were demolded and cured at a temperature of 20 ± 2 °C for 3, 7, 14, 28, and 56 days. The physical properties of the samples were then tested.

#### 2.4.1. Slump

To compare the influence of the treated and untreated coarse RMA on the workability of the concrete mixture, the slump of the fresh concrete mixture was measured following the Chinese specification GB/T 50080-2016 [41]. The slump of the concrete mixture was determined to an accurate value of 1 mm.

#### 2.4.2. Compressive Strength of Concrete

The compressive strength test was carried out on the side of the fractured block. The compressive strength was tested according to the Chinese specification GB/T 17671-1999 [37]. The compressive strength test was carried out at 3, 7, 14, 28, and 56 days.

## 3. Results and Discussion

### 3.1. The Optimal Treatment Method for the o-Bio-Deposition

**Figure 2:**
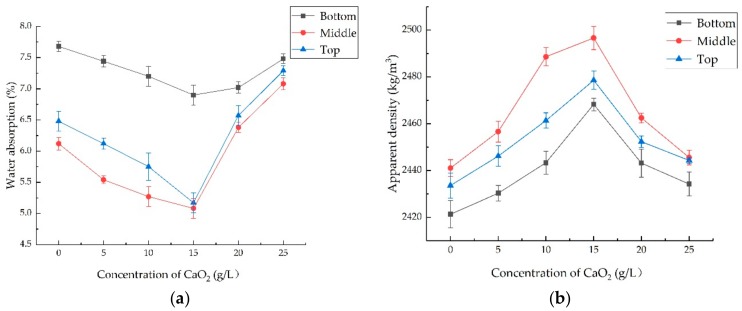

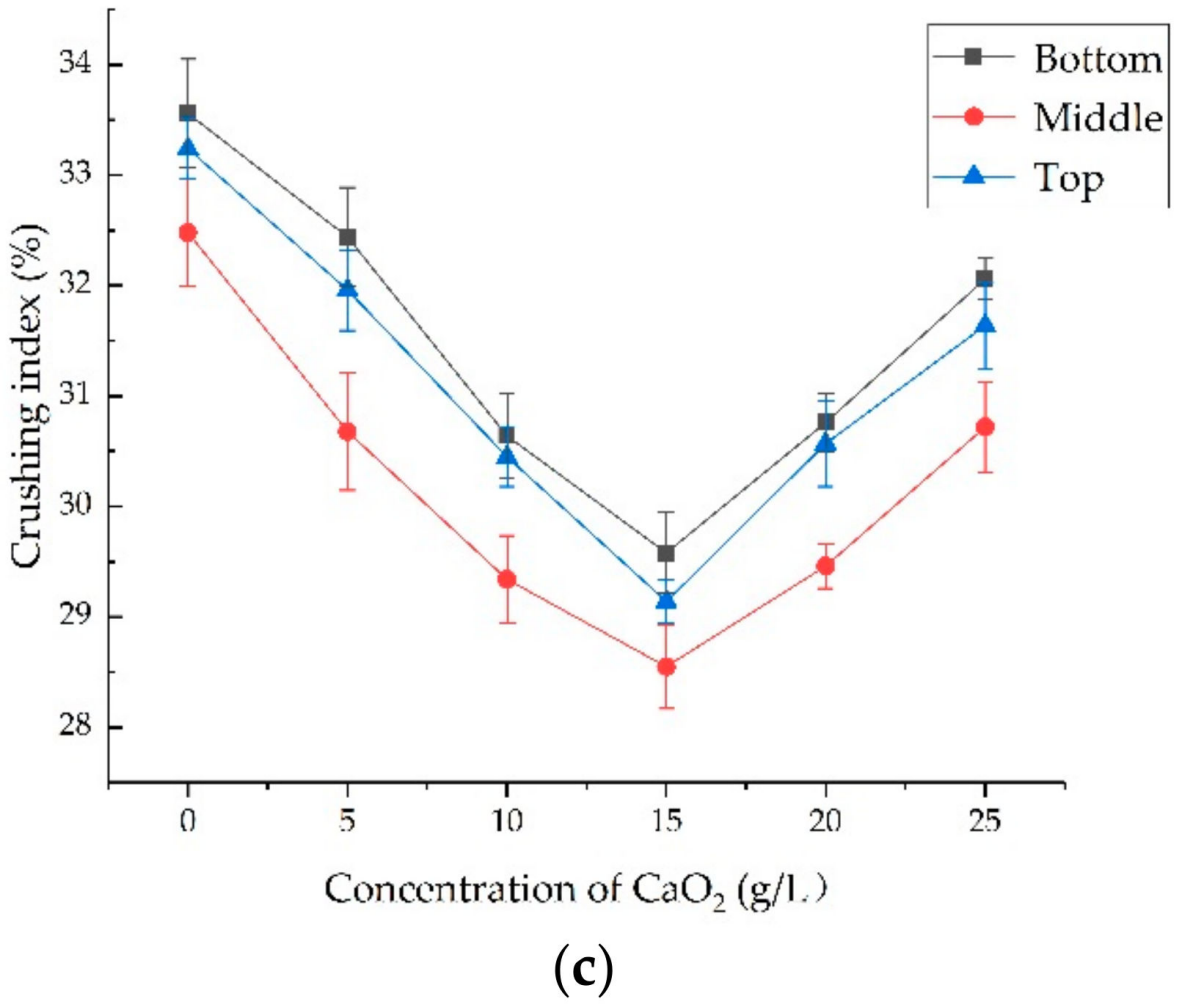
Properties of the treated coarse RMA: (**a**) Water absorption; (**b**) Apparent density; (**c**) Crushing index.

According to the test mentioned in Section 2.2, the properties of the treated coarse RMA were obtained as shown in Figure 2. The results showed that the properties of the coarse RMA were significantly improved by adding CaO_2_ solution to the bacterial solution and changing the immersion position of the coarse RMA compared to the traditional bio-deposition method. When the same concentration of CaO_2_ was added to the culture solution, the modification of the properties of the coarse RMA were optimum in the middle of the immersion position; at the same immersion position, the water absorption and the crushing index of the aggregate first decreased and then increased with the concentration of CaO_2_, and the apparent density increased first and then decreased when the concentration of CaO_2_ was 15 g/L. Thus, we observed the largest improvement in the properties of the coarse RMA.

This may be due to the fact that Bacillus alcalophilus H4 is an aerobic bacteria. There is a lack of O_2_ in the water when treated by bio-deposition; therefore, most of the H4 strain was suspended on the surface of the culture solution to obtain O_2_, which caused the H4 strains to be far from the RMA, and CaCO_3_ precipitation could not effectively accumulate on the surface of the RMA.

As for o-bio-deposition treatment, when the CaO_2_ concentration ranges from 0 to 25 g/L, the addition of 15 g/L of CaO_2_ as an oxygen release compound caused the dissolved oxygen concentration in the water to be the highest [42]. In the presence of oxygen, spores could maintain high metabolic activity, thus improving the ability of aerobic bacteria to produce CaCO_3_. In addition, CaO_2_ reacted with water to produce not only oxygen but also Ca^2+^, which could be used as a supplement to Ca^2+^ for bacteria to induce CaCO_3_ [36]. CaO_2_ could provide O_2_ slowly, which caused the dissolved oxygen rich in the culture solution and the strain H4 was no longer suspended on the surface of the culture solution; instead, it may be because bacteria have a certain weight, the strain H4 accumulated in the middle of the container along with the microcirculation of oxygen in the culture solution. Therefore, when RMA was placed in the middle of the container, strain H4 was closer to the RMA; thus, CaCO_3_ precipitation deposited to the surface of the RMA was more than that of bio-deposition, which affecte thed water absorption, and that in the pores mostly affected the crushing value. The weight of RMA increased in the same volume, thus the apparent density increased.

Comprehensively, when the coarse RMA was immersed in the middle and the concentration of the CaO_2_ was 15 g/L, modification of the RMA was optimal. It was determined that the optimal treatment method was the o-bio-deposition processing method.

**Figure 3:**
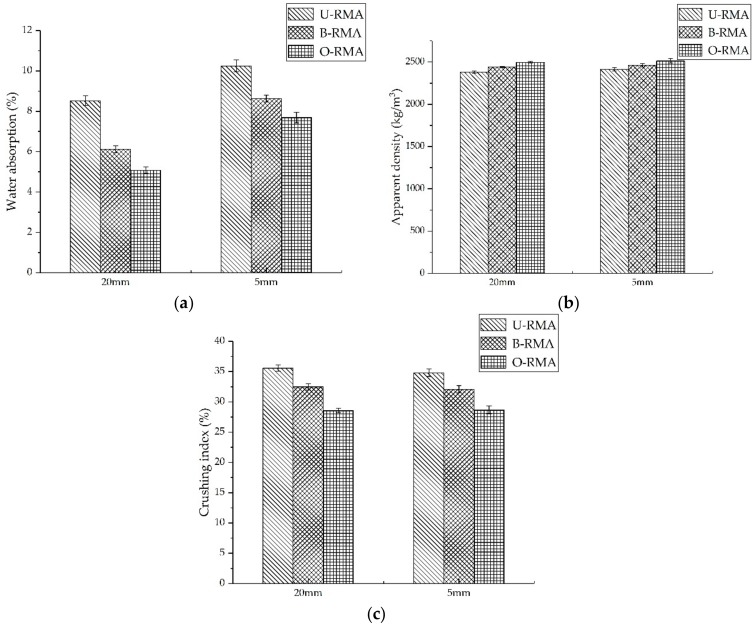
Properties of different particle RMAs: (**a**) Water absorption; (**b**) Apparent density; (**c**) Crushing index.

The fine RMA was treated with o-bio-deposition and bio-deposition. The water absorption, crushing value, and apparent density of the different particle RMAs are shown in Figure 3. The properties of B-RMA and O-RMA improved compared with U-RMA at all size fractions, and the modification of O-RMA was better than that of B-RMA. When the particle sizes of O-RMA were 20 mm and 5 mm, the water absorption of O-RMA was 40.4% and 25%, lower than that of U-RMA, respectively, and those of B-RMA were 28% and 15.8% lower than that of U-RMA, respectively. When the diameter of O-RMA was 20 mm and 5 mm, the crushing indices of O-RMA were 19.8% and 17.8% lower than that of U-RMA, respectively, and those of B-RMA were 8.7% and 7.8% lower than that of U-RMA, respectively. However, the improvement in the apparent density was relatively low. O-RMA and B-RMA decreased by 4.9%, 4.2%, 2.5%, and 2.1% respectively.

From Figure 3, it can be found that the improvement of o-bio-deposition on the properties of RMA at all particle sizes is better than that of bio-deposition. This is because o-bio-deposition not only improved CPA of aerobic bacteria by adding ORC, but also changes the distance between the bacteria and the RMA, so that CaCO_3_ deposited on the surface of the RMA, filling the surface cracks of the RMA, and improving the properties of RMA. Kou et al. [25] reported using CO_2_ to treat RMA and obtained similar results for water absorption when curing 24 h, that is, the water absorption was reduced by 38% for particle of 20 mm. Wu et al. [34] used bio-deposition to treat RCA using aerobic bacteria (DSM8715), the water absorption was reduced by 10% and 23% for particles of 20 mm and 5 mm, and the crushing value reduced by 15% and 12% for the particle sizes of 20 mm and 5 mm. Compared with previous studies, it can be found that the performance of RMA improved by o-bio-deposition was similar to that by CO_2_ treatment. Moreover, the modification of bio-deposition using strain H4 on recycled aggregate is better than that of DSM8715. This is because different aerobic bacteria have different CPA (CPA of the strain H4 is higher than DSM8715) [35].

**Figure 4:**
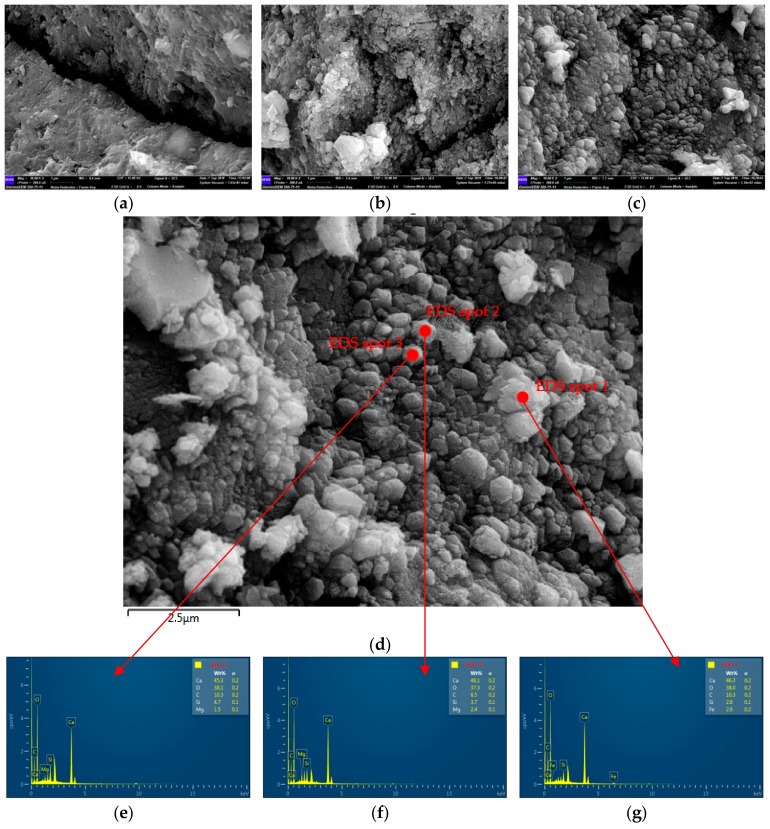
SEM-EDS images of the RMA surface: (**a**) the U-RMA surface; (**b**) the B-RMA surface; (**c**) the O-RMA surface; (**e**–**g**) EDS spectra of the particles in (**d**).

The surface of the untreated and treated RMA was observed by SEM, and the chemical composition of the deposited particles on the surface of RMA was determined by EDS (energy dispersive spectroscopy) (Figure 4). In the three specimens (Figure 4d), the particles were CaCO_3_ as indicated by EDS analyses (Figure 4e–g). There was no CaCO_3_ on the surface of the untreated RMA (Figure 4a). Calcite-type CaCO_3_ crystals were produced on the surface, as well as cracks of B-RMA (Figure 4b); however, they were not enough to fill the micro-cracks. Compared with B-RMA, several CaCO_3_ crystals were observed on the surface and the cracks of the O-RMA to completely fill the micro-cracks (Figure 4c,d).

### 3.2. Properties of Concrete

#### 3.2.1. Slump of Concrete Mixtures

**Figure 5:**
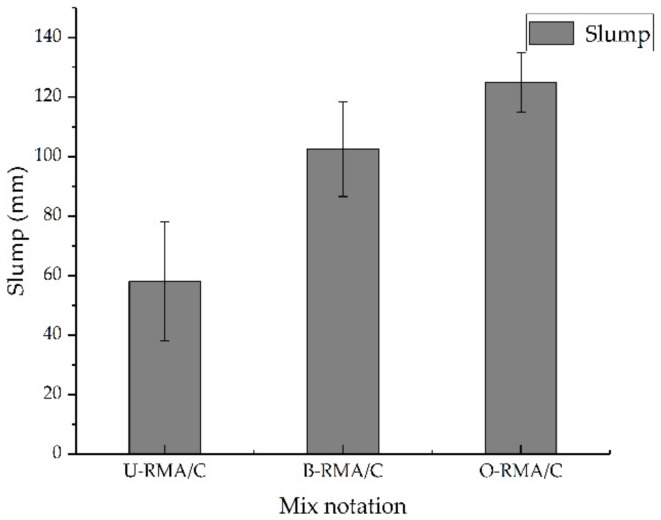
Slump of fresh concrete.

Figure 5 shows the results of the slumps of the concrete mixtures. The reported values are the average of three measurements. The slump value of B-RMA/C and O-RMA/C increased compared to that of U-RMA/C, and O-RMA/C has the largest slump (125 mm). The slump value of U-RMA/C is 58 mm with an increase in B-RMA/C relative to U-RMA/C of 70.7%, and that of O-RMA/C relative to U-RMA/C is 115.5%. This may be due to the decrease in water absorption of the treated aggregate B-RMA and O-RMA, resulting in an increase in free water content in the concrete mixtures.

#### 3.2.2. Compressive Strength

**Figure 6:**
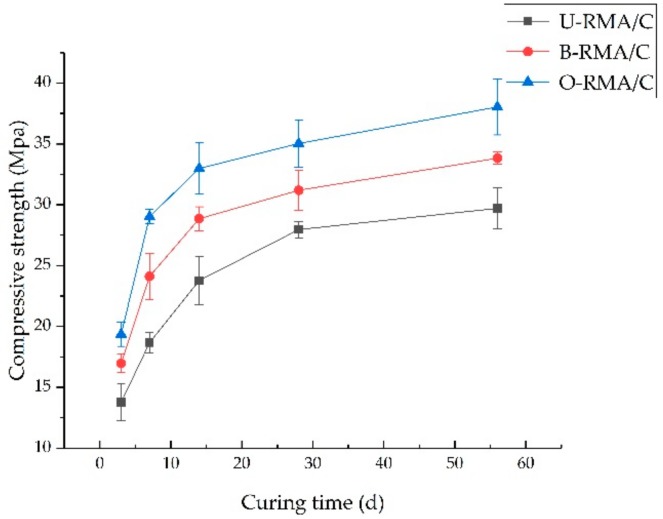
Compressive strength of concrete.

The compressive strength of concrete developed until 56 d, as shown in Figure 6. The compressive strength of O-RMA/C was higher than that of B-RMA/C and U-RMA/C during all the testing periods. The compressive strength of the cube was 13.76 MPa at 3 d of U-RMA/C. The compressive strength of B-RMA/C and O-RMA/C at 3 d increased by 23.18% and 40.70% compared with U-RMA/C, respectively. At 7 d, 14 d, 28 d, and 56 d, the compressive strength of B-RMA/C increased by 29.2%, 21.3%, 11.6%, and 13.9%, respectively, whereas that of the -RMA/C increased by 55.5%, 38.7%, 25.3%, and 28%, respectively.

The improvement of compressive strength may be attributed to bio-deposition treatment. CaCO_3_ produced by bacteria plays a positive role in improving the compressive strength, which improves the physical properties of B-RMA and O-RMA. As shown in from Figure 3, the water absorption of 20-mm O-RCA and B-RCA is 40.4% and 28% lower than that of U-RCA, and the crushing index of the O-RCA and B-RCA is 25% and 15.8% lower than that of the U-RCA, respectively. The physical properties of O-RMA are better than that of B-RMA. Figure 4 shows that the O-RMA was covered with more CaCO_3_ particles than B-RMA. Fine CaCO_3_ promotes a hydration reaction and enhances the ITZ (interfacial transition zone) [43]; thus, the compressive strength of O-RMA/C is higher than that of B-RMA/C. The compressive strength of the B-RMA/C and O-RMA/C increased more than that of the U-RMA/C from 3 to 7 d, and the increase was similar with the mass death of bacteria in the later period.

#### 3.2.3. SEM Analysis of Micro-Cracks in Concretes

**Figure 7:**
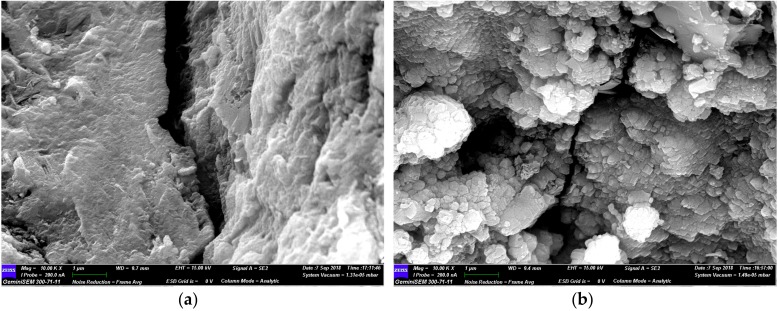
SEM images of micro-cracks in concretes: (**a**) U-RMA/C and (**b**) O-RMA/C.

The micro-cracks of the concretes can be observed in Figure 7. There are evident cracks in the U-RMA/C (Figure 7a), and some hydration products near the cracks; however, there are not enough to fill and cover the cracks. There are also cracks in the interface transition zone of O-RMA/C (Figure 7b); however, the width of the cracks is evidently narrow, and several hydration products near the cracks fill the cracks effectively. Hydration products exist in clusters near the cracks. These may be attributed to the fine calcium carbonate particles that can provide nucleation sites for the hydration products, thus promoting the hydration reaction for increasing the hydration products to fill the micro-cracks in the concrete and improve the compressive strength of concrete. [43].

## 4. Conclusions

1. Upon comparing the properties of RMA under different treatment methods, we determined that the treatment method of o-bio-deposition involves immersing the aggregates in the middle of the container and adding 15 g/L CaO_2_ to the bacterial solution of Bacillus alkalophilus H4. The water absorption and crushing index of 20-mm O-RMA are 40.4% and 19.8% higher than those of U-RMA, respectively. The properties of the O-RMA are superior to that of the B-RMA.
2. SEM-EDS showed that there are many pores and micro-cracks in the U-RMA, the width of which vary from 0.02 µm to 1.2 µm. The particles on the surface of the O-RMA were CaCO_3_ as indicated by EDS analyses. Several CaCO_3_ crystals could be seen on the surface and cracks of the O-RMA that completely fill the micro-cracks.
3. The compressive strength and slump of the B-RMA/C and O-RMA/C increased compared with U-RMA/C, and the increase in compressive strength of the O-RMA/C is 25.3% at 28 d. In addition, according to the SEM images of the micro-cracks of concrete, it was found that the micro-cracks of the O-RMA/C were filled by hydration products, and improved the compressive strength of concrete.

## Figures and Tables

**Table 1:** Proportions of concrete mixtures.

Notation ^1^	Proportion (kg/m^3^)					
	w/c Ratio	Sand Ratio	Cement	Fine Aggregates	Coarse Aggregates	Water
U-RMA/C	0.5	40	370	738	1107	185 + 36.7
B-RMA/C	0.5	40	370	738	1107	185 + 22.1
O-RMA/C	0.5	40	370	738	1107	185 + 14.7

^1^: U-RMA/C: the concrete made from untreated coarse RMA; B-RMA/C: the concrete made from RMA treated by bio-deposition; O-RMA/C: the concrete made from RMA treated by o-bio-deposition.
